# Does the patellar tendon reflex affect the postural stability in stroke patients with blocked vision?

**DOI:** 10.1515/tnsci-2022-0283

**Published:** 2023-04-17

**Authors:** Ziyou Zhou, Zhen Hu, Wei Bao, Ying Yang, Kai Chen

**Affiliations:** Department of Mechanical Engineering, School of Mechanical Engineering, Hangzhou Dianzi University, No.1158, Xiasha 2nd Street, Jianggan District, Hangzhou, Zhejiang 310018, China; Department of Neurology, Ruijin Hospital Affiliated to Shanghai Jiao Tong University, Shanghai 200000, China

**Keywords:** tendon reflex, balance control, centre of pressure, detrended fluctuation analysis, power spectral density

## Abstract

**Background:**

Stroke patients often show postural instability. The patellar tendon reflex is a basic physical examination for stroke patients. This study aimed to explore the correlation between patellar tendon reflex grade and postural stability among stroke patients.

**Methods:**

A total of 37 elderly stroke patients, each with the same quadriceps muscle strength but different patellar tendon reflex levels, were tested on a force platform under eyes-open (EO) and eyes-closed (EC) conditions. Parametric analysis, detrended fluctuation analysis (DFA), and power spectral density (PSD) analysis were used in centre of pressure (COP) signal processing. The correlation between the results of measured data processing and the level of patellar tendon reflex was analysed.

**Results:**

All three parameters of COP (the length of the sway trajectory, the mean range of the sway trajectory in the mediolateral [ML] direction [*R*_*x*_], and the mean range of the sway trajectory in the anterior–posterior [AP] directions [*R*_*y*_]) were negatively correlated with the patient’s patellar tendon reflex grade under the EC condition. The DFA results showed that a higher grade of patellar tendon reflex was associated with a smaller value of the crossover point in the AP direction. Only the PSD values of each frequency band in the AP direction were negatively correlated with patellar tendon reflex grade with EO and became negatively correlated in both AP and ML directions with EC. Overall, the results showed a strong correlation between patellar tendon reflex and postural stability in stroke patients when vision was blocked.

**Significance:**

The strong correlation with EC may provide insights into clinic evaluation and treatment for rehabilitation or fall risks of stroke patients.

## 1 Introduction

Stroke is a serious threat to human health. The adverse consequences of a stroke can include muscle strength decline, coordination loss, and postural instability [1,2]. Impaired postural stability after stroke may greatly impact gait and self-care ability, and is also the main reason for falls in stroke patients. At the same time, it will increase the burden of care [3]. Therefore, improving the balance of stroke patients is an important component of rehabilitation [4].

Posture and balance are maintained by the coordination of various sensory systems, including the visual system, the vestibular system, the somatosensory system, muscle spindles, and the central nervous system [2]. The lower limb joints and their surrounding muscle tissues play an important role in postural stability because the joints and muscles provide a sense of position and motion of the limbs [5]. Tendon reflexes are monosynaptic reflexes in the body [6]. The muscle spindle is the proprioceptor oriented parallel to the extrafusal fibres of skeletal muscles to maintain the length monitoring system, which is essential for postural balance. Together, they provide a negative feedback loop that maintains muscle length and posture [7]. Impaired synaptic transmission can lead to limb stability disorders [6].

Assessing tendon reflexes is an effective means of evaluating the function of the nervous system in stroke patients. The amplitude of tendon reflexes depends on the excitability of the α motor neuron pool in the spinal cord [8]. The reflex excitability and its amplitude can reflect the health status of the central and peripheral nervous systems [9]. This reflex can be observed when tapping the tendon of the quadriceps femoris in stroke patients. The strength of the knee patellar tendon reflex is related to many physiological indicators, such as functional changes in the neuromuscular system [10], predicting improvement in patients with motor paralysis [11], and an improved sense of joint position in hyperreflexia [12].

At present, the assessment of human postural stability is often achieved by measuring postural sway while standing on a force platform [13]. The real-time displacement of a subject’s centre of pressure (COP) can be achieved through the force platform. Global COP parameters, such as the length and range of sway trajectory, are commonly used to evaluate postural stability. However, these parameters give few insights into the control of posture. Different signal processing methods have been developed from nonlinear theories in order to retrieve more information on the mechanisms of postural control. Among them, detrended fluctuation analysis (DFA) is one of the most applied methods [14,15,16,17]. It describes the signal as persistent when there is a positive correlation with time and anti-persistent when there is a negative correlation with time. Alternatively, the COP coordinates can be decomposed and analysed in terms of rambling (RA) and trembling (TR) [18]. In relationship with the DFA, the persistent region can capture the TR components while the anti-persistent region reflects the RA components [19]. Power spectral density (PSD) analysis is another powerful method to analyse the frequency-domain characteristics of signals [20,21,22]. It helps to find sensitivities of complex attributes associated with physiological systems and leads to detecting and discriminating postural control system impairments [21].

Quadriceps muscle strength and the patellar tendon reflex may be related to postural stability and hence the risk of falls. For example, Paillard and Borel [23] found that both unilateral and bilateral muscle fatiguing contractions of quadriceps femoris reduce posture stability, and that unilateral muscle fatigue creates postural asymmetries. Shelbourne et al. [24] have shown a significant correlation between the width of tendons and muscle strength. Like muscle strength examination, the patellar tendon reflex is also the most basic physical examination conducted with stroke patients [25]. However, its correlation with postural stability has not been studied. In addition, whether the patellar tendon reflex is associated with postural stability under different sensory conditions has not been investigated.

The current study explored the correlation between the patellar tendon reflex and postural stability in stroke patients with or without visual feedback while controlling for muscular strength. The COP coordinate data were further analysed by the aforementioned signal processing methods to reveal more detailed information on the postural stability of stroke patients. The authors believe that this study provides insights into the control mechanisms for postural stability regulation, and the research results will be helpful for the rehabilitation of stroke patients by detecting and reducing the risk of falls.

## 2 Materials and methods

### 2.1 Participants

A total of 37 elderly stroke patients participated in this study, including 20 men and 17 women, with an average age of 63 years (54–70 years). At the same time, seven healthy participants matched by gender, height, and age were selected as the control group. The participants’ clinical and demographic features are presented in Table 1. The inclusion criterion envelope for participants is as follows: (1) stroke was diagnosed separately by two neurologists who had been practicing for more than 10 years. (2) The time of the first disease was within 1 year, and all were in the early stage of the disease course. (3) Their quadriceps muscle strength grades were all V (each stroke patient was diagnosed independently by two physicians with more than 10 years of clinical experience. If both physicians considered the patient to be Grade V, the patient was included in the study). The manual assessment was used to measure the quadriceps muscle strength of patients, where Grades 0, I, II, III, IV, and V were used to classify the strength. Grade V indicates that muscle is normal and movement is free. (4) The participants have the ability to understand instructions. (5) The participants are interested in participating in the study. Exclusion criteria are as follows: (1) all patients with unconsciousness and a history of surgery; (2) patients with lesions (cerebellar, brain stem, parietal) that affect balance and posture; (3) those tested on the H&Y scale at a rating of 4–5; (4) patients with significant cognitive impairment, pacemakers, coronary stents, and cochlear implants; (5) patients who have been treated with drugs that affect their balance; (6) patients diagnosed with orthopaedic conditions such as arthritis, fractures, and lower back pain; and (7) patients with vision problems such as glaucoma, cataracts and diplopia.

**Table 1:** Clinical and demographic features of the patients

Variable	Men (*N* = 20)	Women (*N* = 17)	Control (*N* = 7)
Age, years	63.6 ± 4.9	62.4 ± 4.7	62.8 ± 3.5
Body weight, kg	68.1 ± 6.4	54.4 ± 6.3	58.2 ± 7.2
Side of hemiparesis, left/right	15/5	14/3	N/A
Haemorrhage/infarction	3/17	2/15	N/A
Disease duration, days	38.5 ± 7.4	37.6 ± 5.6	N/A
BBS	46.9 ± 3.0	47.4 ± 3.9	54.1 ± 1.4

BBS, Berg balance scale. Data are provided as mean ± standard deviation unless otherwise noted.


**Informed consent:** Informed consent has been obtained from all individuals included in this study.
**Ethical approval:** The research related to human use has been complied with all the relevant national regulations, institutional policies and in accordance the tenets of the Helsinki Declaration, and has been approved by the authors' institutional review board or equivalent committee. Research was was approved and supervised by the ethics committee of the Luwan branch of Shanghai Ruijin Hospital (approval No.: lwech219017).

### 2.2 Grading of the patellar tendon reflex

**Figure 1:**
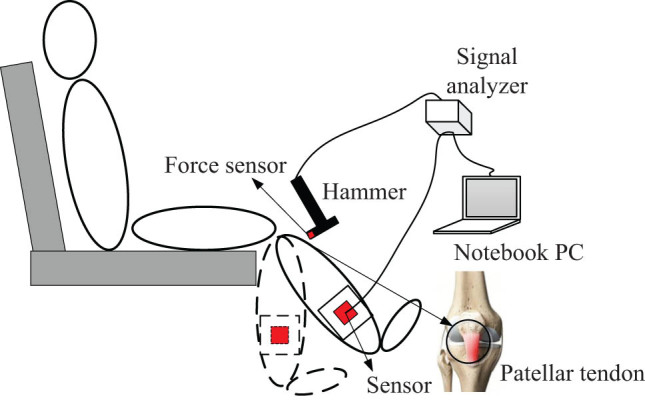
Experimental setup for patellar tendon tapping. The subject sits on a high stool with both feet not touching the ground. A tri-axial accelerometer is fixed on the short leg brace at the medial malleolus. To allow the operator to hit each subject equally hard, a force sensor is mounted on the tip of the hammer. The patellar tendon reflex rating can be read on a laptop computer.

Although it has been reported that the velocity and latency are important indicators for the patellar tendon reflex [26], the magnitude is still used to evaluate the reflex arc in most clinical practices. The current study followed the traditional method and also used the amplitude. The National Institute of Neurological Disorders and Stroke has developed a five-grade grading standard. When the tester’s level is (−), it means that the reflection disappears; (+) means that the reflection is lower than normal; (++) means that the reflection is within the normal range; (+++) means that the reflection is slightly higher than the normal range; (++++) means that the reflection is significantly higher than the normal level. We rated the subjects’ knee tendon reflexes according to the above standard, with the detailed method described in the study of Mamizuka et al. [27]. Patellar tendon reflex tests were performed independently for each participant by two physicians with more than 10 years of clinical experience. As shown in Figure 1, the patient was asked to sit upright in a reclining chair fitted with a hammer that was used to hit the knee tendon with a certain amount of force. The tapping force was measured using a push–pull force meter (ZTA-500N; Imada Inc., Japan) to control the consistent force of each tapping; the experimenter can control the force so that it is the same for each patient. The results can be read on the computer. The 37 stroke patients’ scores of the patellar tendon reflex were divided into five grades: ++++ spasm (2), +++ active (3), ++ normal (18), + decreased (8), and – disappeared (6). Seven participants of the control group were all in the ++ grade. The number in parentheses indicated the number of patients in the corresponding grade. Patients with the same score on left and right limbs were included as the experimental sample of this study. Assessment of the patellar tendon reflex was conducted by two independent doctors with extensive clinical experience, and patients with different results were excluded.

### 2.3 Measurements of the COP

**Figure 2:**
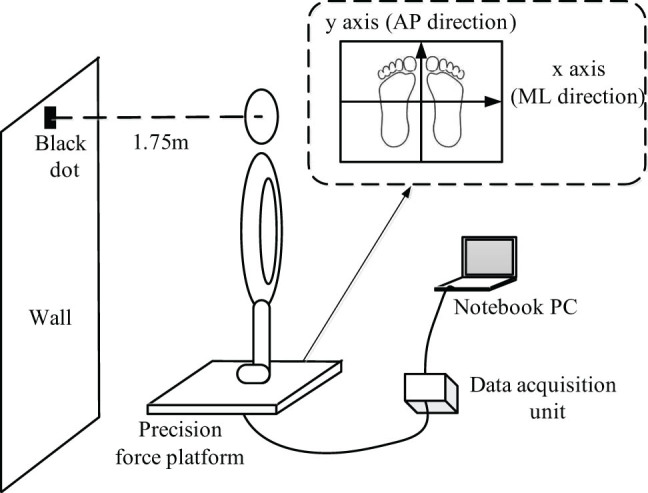
Experimental details of the real-time coordinates of the participant’s COP.

In this study, a precision force platform (Model BP400600; Advanced Mechanical Technology Inc., MA, USA) was used to measure the real-time coordinates of the participants’ COP. Before the experiment, the requirements and precautions were explained to the patients in detail. As shown in Figure 2, the patients were asked to stand still on the platform with their feet shoulder-width apart and their hands naturally drooping along the side of the body, while looking at a small target in front. During the eyes-open (EO) test, the subject’s gaze was fixed on a 5 cm diameter black dot pasted on the wall 1.75 m height above the ground and meters from the subject. The test environment was kept quiet. The patients were also asked to wear a blind-fold under the eye-closed (EC) condition.

The participants were asked to maintain their standing posture for 30 s before the recording of the COP coordinates started and continued for a further 40 s. The same process was repeated for each condition (EO and EC) with at least 5 min for participants to relax between each test. Data for the first and the last 5 s were removed to avoid any disturbances at the beginning and end. The sampling frequency of the pressure transducer was 500 Hz. In data processing and calculation, the *x*-axis is the subject’s mediolateral (ML) direction, which is parallel to the frontal plane. To maintain the reliability of the collected data, each patient’s test was repeated three times, and the average value was taken during the parameter calculation.

### 2.4 Data pre-processing

#### 2.4.1 Step 1. Analysis of the COP parameters

The experimental data were analysed using MATLAB® (R2017a; The MathWorks Inc., Natick, MA, USA). In this study, postural stability was assessed by the COP length of the sway trajectory (represented as LST) and the mean range of the sway trajectory in the *x*-axis (ML) and *y*-axis (AP), expressed as *R*_*x*_ and *R*_*y*_, respectively.

#### 2.4.2 Step 2. Analysis with the DFA method

The DFA of the velocity signal of the COP trajectory was based on the method described by Zhou et al. [19]. The values of *α*_1_ (short-term scale index), *α*_2_ (long-term scale index), and the crossover point (CP) were calculated and counted.

#### 2.4.3 Step 3. Analysis with PSD

PSD presents the strength variation of the signal as a function of frequency. The computation of PSD is done directly by the well-known fast Fourier transform (FFT) method [28]. FFT is programmed and calculated by MATLAB^®^. According to the literature [18,29], the frequency of the RA signal is concentrated at about 0–0.5 Hz, while that of the TR signal is concentrated at about 0.5–1.5 Hz. The influence of the TR signal on the human body balance is mainly caused by the ankle joint and hip joint. The swing frequency of the ankle joint is concentrated between 0.5 and 1 Hz, and that of the hip joint is concentrated between 1 and 1.5 Hz. So, here the PSD of the COP sway signal was calculated, and the average PSD values of frequency ranges 0–0.5, 0.5–1.0, and 1.0–1.5 Hz were counted.

#### 2.4.4 Step 4. Statistical analysis

IBM SPSS statistics 25.0 was used for statistical analysis. Spearman correlation analysis was used to calculate the correlation. The significance level of all statistical analyses was set as *P* < 0.05.

## 3 Results

According to the COP trajectory parameters in Table 2, the LST and the mean ranges of the sway trajectory of COP in the ML and AP directions (*R*_*x*_ and *R*_*y*_, respectively) of the control group were all lower than those of the test group. In the group, under the condition of natural standing with EO, *LST* was significantly and negatively correlated with the patellar tendon reflex grade (*P* = 0.050; *r* = −0.325). *R*_*x*_ (*P* = 0.293; *r* = −0.178) and *R*_*y*_ (*P* = 0.293; *r* = −0.38) had no significant correlation with the patellar tendon reflex grade. However, under the condition of natural standing with EC, there were significant negative correlations between all three parameters of COP and patellar tendon reflex (LST, *P* = 0.001, *r* = −0.528; *R*_*x*_, *P* = 0.021, *r* = −0.378; *R*_*y*_, *P* = 0.001, *r* = −0.628). This indicates that the patellar tendon reflex level of stroke patients is associated with postural stability, and this correlation enhances when visual feedback is removed.

**Table 2:** COP LST and mean range radius in ML (*R*_*x*_) and AP (*R*_*y*_) directions, the significant difference values and correlation coefficients with patellar tendon reflex grades of stroke patients

	EO			EC			Control
	Mean	*P*-value	*r*	Mean	*P*-value	*r*	Mean
*LST*	97.2	0.050*	−0.325	104.3	0.001**	−0.528	69.5
*R*_*x*_	3.0	0.293	−0.178	3.6	0.021*	−0.378	2.1
*R*_*y*_	3.1	0.293	−0.38	2.7	0.001**	−0.628	2.4

Note: * significant under *P* = 0.05. ** significant under *P* = 0.01.

**Figure 3:**
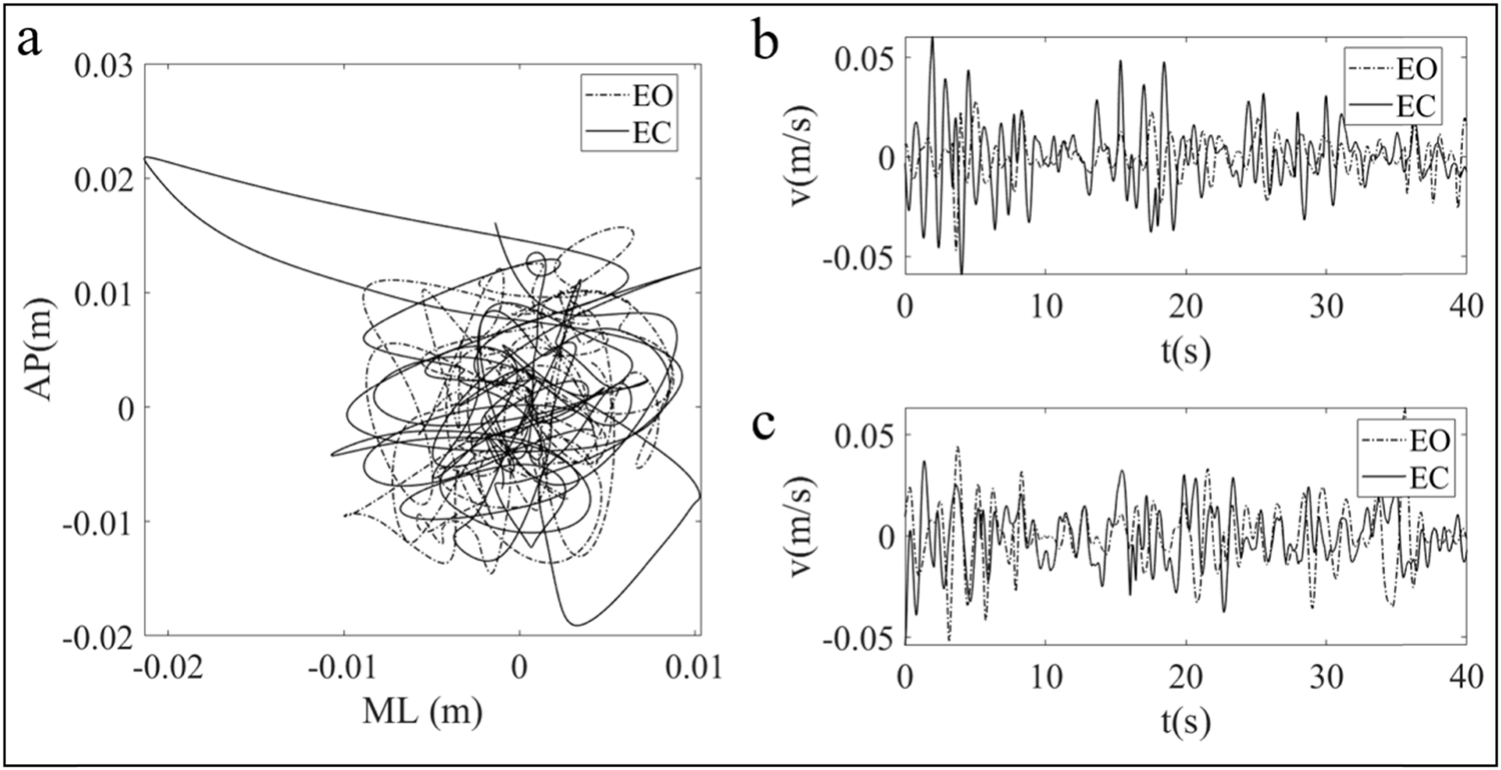
(a) The typical COP sway trajectories under the conditions of EO and EC. (b) Time sequential diagrams of COP sway velocity in the AP direction with EO and EC. (c) Time sequential diagrams of COP sway velocity in the ML direction with EO and EC.

**Figure 4:**
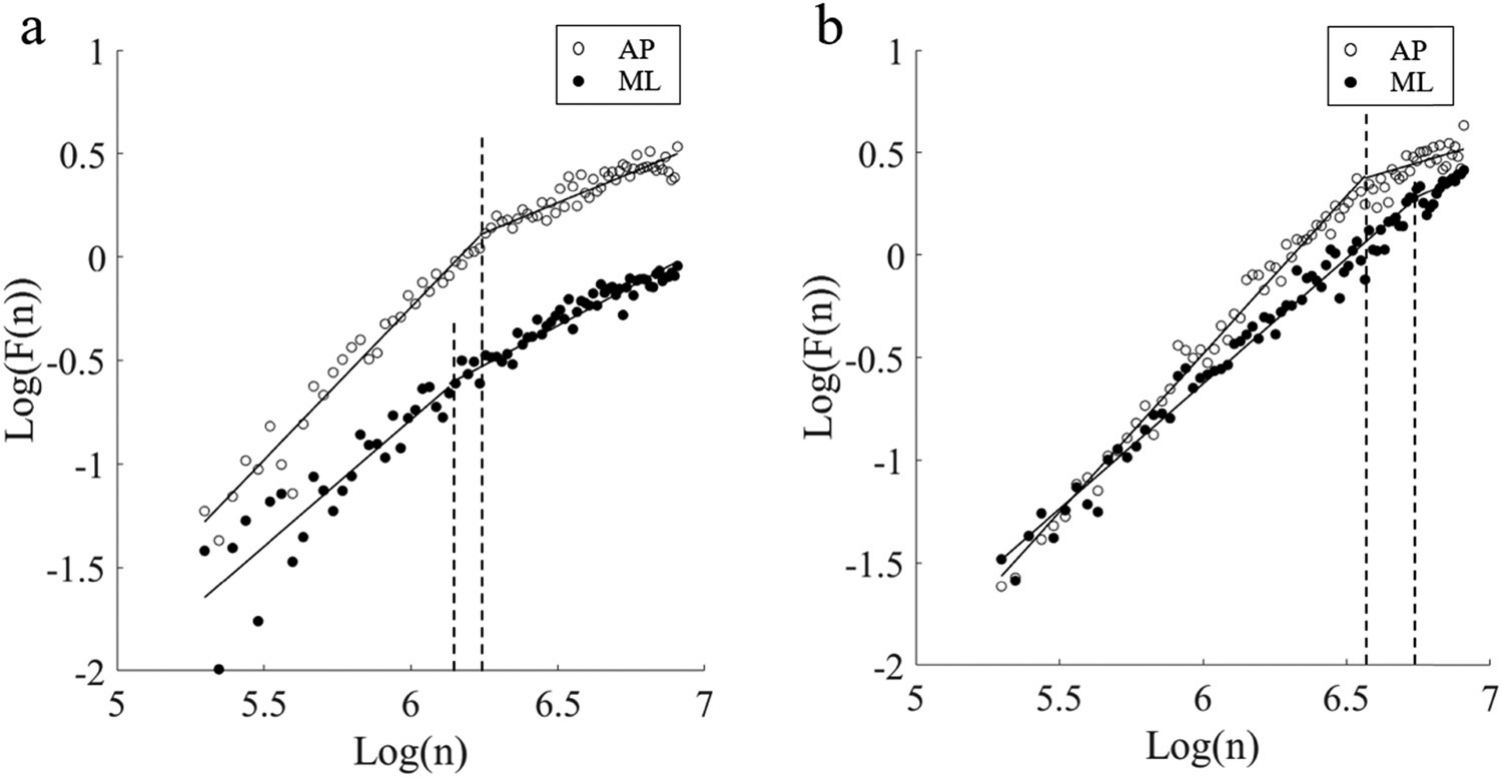
Typical logarithmic diffusion diagrams generated by DFA of COP sway velocity. (a) EO. (b) EC.

**Figure 5:**
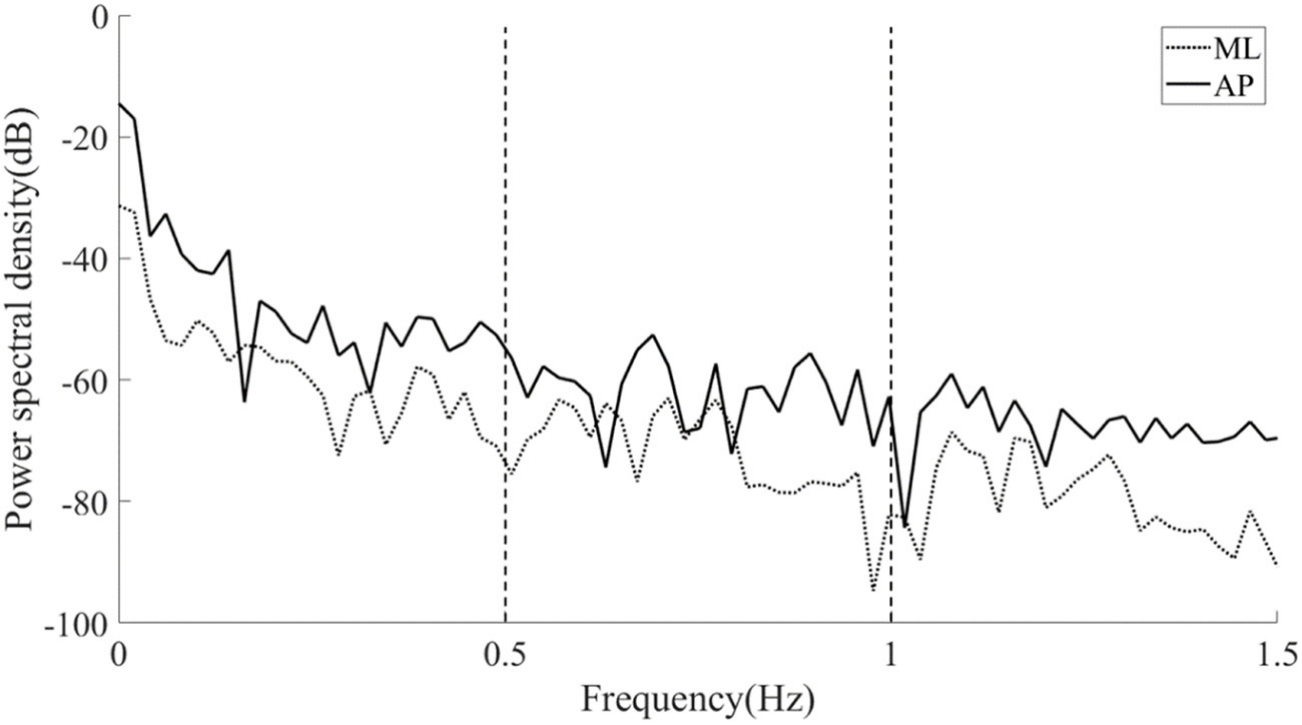
PSD analysis diagram of a typical COP position signal.

Figures 3–5 are drawn based on the same individual data of the test group and represent the typical characteristics of the whole data. Figure 3a shows the two sway trajectories of a patient’s COP on the coordinate axis with EO and EC. Figure 3b and c shows the time series diagrams that comprise Figure 3a into the sway velocity in the AP and ML directions, respectively. After the time series of the COP velocity were obtained, the DFA was applied, and the log–log diffusion diagram is presented in Figure 4. The fitting result can be regarded as composed of two straight lines and a CP. The slope of the left line over the CP is greater than 1, and the slope of the right line is less than 1. This means that the COP sway velocity is a persistent signal with a positive correlation in the short term and becomes an anti-persistent signal with a negative correlation in the long term. The results are in accordance with the law of postural regulation, as confirmed in many previous studies [15].

Figure 5 depicts the results of PSD analysis on a typical COP recording in the ML and AP directions. The value of PSD on the longitudinal axis represents the energy of postural sway. The local sway energy was obtained by comparing the PSD values of each frequency band. The absolute PSD value of postural sway in the AP direction was greater than in the ML direction, and the sway energy decreased as the frequency band shifted from 0–0.5 to 0.5–1.0 Hz and further to 1.0–1.5 Hz.

Table 3 shows the statistical results of both DFA and PSD analyses of the COP signals under the condition of EO. There was no significant correlation between the DFA fractal parameters in the ML direction with the patellar tendon reflex grades of the testers. There were also no significant correlations between the mean PSD values in all three different frequency bands with the patellar tendon reflex grades. However, patellar tendon reflex grade was negatively correlated with the mean CP value of DFA results in the AP direction (*P* = 0.021; *r* = −0.377), and also the mean PSD values of all three frequency bands in the AP direction, specifically, in fb1 (0–0.5 Hz frequency band; *P* = 0.001; *r* = −0.560), in fb2 (0.5–1.0 Hz band; *P* = 0.022; *r* = −0.376), and in fb3 (1.0–1.5 Hz band; *P* = 0.003; *r* = −0.474).

**Table 3:** Mean values from DFA and PSD analyses of COP signals, and the significant difference values and correlation coefficients with patellar tendon reflex grades of stroke patients with EO

EO	ML direction			AP direction		
	Mean	*P*-value	*r*	Mean	*P*-value	*r*
CP	4.89	0.192	−0.219	0.05	0.021*	−0.377
fb1	−49.46	0.106	−0.27	−48.08	0.001**	−0.56
fb2	−57.58	0.702	−0.065	−55.975	0.022*	−0.376
fb3	−66.55	0.482	−0.119	−65.067	0.003**	−0.474

Note: * significant under *P* = 0.05. ** significant under *P* = 0.01.

Table 4 shows the statistical results of the COP signals measured in the EC state after DFA and PSD analysis. During the state of EC, the mean PSD values of all three frequency bands were negatively correlated with the patellar tendon reflex grades. Specifically, in the range of fb1 (*P* = 0.021; *r* = −0.379), fb2 (*P* = 0.043; *r* = −0.334), and fb3 (*P* = 0.045; *r* = −0.322). The CP value of DFA results in the AP direction (*P* = 0.001; *r* = −0.663), and the mean PSD values of all three frequency bands in the AP direction were significantly negatively correlated with patellar tendon reflex grades, specifically, in the range of fb1 (*P* = 0.007; *r* = −0.436), fb2 (*P* < 0.001; *r* = −0.662), and fb3 (*P* = 0.001; *r* = −0.643).

**Table 4:** Mean values DFA and PSD analyses of COP signals, and the significant difference values and correlation coefficients with patellar tendon reflex grades of stroke patients with EC

EC	ML direction			AP direction		
	Mean	*P*-value	*r*	Mean	*P*-value	*r*
CP	−0.34	0.087	−0.285	−0.08	0.001**	−0.663
fb1	−51.66	0.021*	−0.379	−48.25	0.007**	−0.436
fb2	−59.26	0.043*	−0.334	−56.91	0.001**	−0.662
fb3	−68.31	0.045*	−0.332	−66.14	0.001**	−0.642

Note: * significant under *P* = 0.05. ** significant under *P* = 0.01.

Overall, the patellar tendon reflex level of stroke patients is associated with representative COP variables which represent postural stability, particularly in the AP direction, and the correlation becomes significant under the condition of blocked vision.

## 4 Discussion

This study explored the correlation between stroke patients’ patellar tendon reflex grade and their postural stability. The patellar tendon reflex was graded by the traditional method of knocking and recording, while postural stability was measured and analysed by the patient’s COP data collected from a precision force platform. The experimental design eliminates the effects of the muscular system as an actuator in postural stability regulation by selecting patients with the same quadriceps muscle strength, thereby improving the efficacy of the study conclusions.

The statistical results of the COP parameters showed that the patellar tendon reflex grade was somehow negatively related to the postural stability of stroke patients with and without visual feedback. The correlation was improved to significant levels after visual feedback was removed (all three parameters). Since the global COP parameters obtained from the statokinesigram are commonly used to assess the human’s postural stability, with smaller values indicating better balance control, the results show that the patellar tendon reflex is effectively associated with stroke patient’s postural stability when the visual system is blocked.

Compared with the traditional COP index, the results from the DFA method reflect the dynamic processes of postural control [30]. The results showed that the CP in the AP direction had a negative correlation with the tendon reflex level when visual feedback was available, and the correlation was also improved to significant without visual feedback. The corresponding slope values of two regression lines (*α*_1_, *α*_2_) can be used to represent the characteristics of the short-time persistent and long-time anti-persistent signals. The postural control system adopts an open-loop control scheme associated with persistence, while a closed-loop control scheme is associated with anti-persistent signals [11]. It was reported that the fast-scale persistent region captures the TR component that deviates around a reference frame, which is associated with the elementary spinal and muscular reflex, while the anti-persistent slow-scale region indexes the RA dynamics, which sets the reference and is related to central nervous system modulation [30]. Recently, the theory of intermittent control has arisen as an attractive supplemental mechanism for postural control. It is suggested that ballistic, pre-programmed signal provides the slow-scale central executive control driving fast-scale, continuous feedback inner control loops [23,29,31,32]. Particularly, Elias et al. [29] reported that posture control might be partly mediated by spinal mechanisms, with proprioceptive information being fed back to the spinal neuronal circuitry.

The patellar tendon reflex is a phasic stretch reflex, a quick motor neuron discharge caused by brief stimulation of muscle spindles or their afferent nerve pathways. The size of CP represents the sensitivity of postural stability adjustment, that is, the threshold value for intermittent active postural regulation [16]. The smaller the CP values, the more the sensitivity of regulation. It can be inferred that the decrease of patellar tendon reflex grade results in an impaired phasic response and thus less sensitive or more latency of feedback inner control, which corresponds to a larger CP value. Visual input has predominantly influenced corrective responses for the control of AP sway in all bandwidths [21]. The afferent pathway of vision involves higher centres of CNS. The patellar tendon reflex only travels back to the spinal cord and synapses at the lower levels. When the vision is blocked, one has to rely more on the somatosensory system for postural control, and the patellar tendon reflex becomes more important. This is reflected by a strong correlation of CP value to the grade of patellar tendon reflex when visual input is blocked.

Analysis with PSD transfers the time series of the signal into frequency domains. Larger PSD values indicate more energy of postural sway and reduced control capability of the subject [21]. The current results showed that the mean PSD values of the three frequency domains in the AP direction were negatively correlated with patellar tendon reflex with or without visual feedback, while in the ML direction, they were only relevant when visual feedback was removed. The correlation coefficients of the high bands in the AP direction were also improved when visual was not available. The absolute value of PSD decreases when the frequency band shifts from a lower to a higher frequency, which is in agreement with previous findings [1], inferring a higher ability of the fast scale control compared to the slow scale control. The absolute PSD value increases when the visual system is blocked at the same frequency band, resulting in less agile control mechanisms. The correlation between PSD and patellar tendon reflex was more significant when the eyes were closed, which is the same outcome as obtained from the DFA.

The aforementioned results from the global descriptors of stabilogram and from DFA and PSD indicate that the patellar tendon reflex is a better reflection of postural stability in the AP direction than in the ML direction. This is attributed to the fact that the ankle strategy, as the primary stabilizer, predominantly regulates sway in the AP direction, whereas the hip strategy regulates ML sway [21]. Participants were tested in two states (EO and EC) to explore the interactive effects between vision and the central nervous system in controlling posture. The experimental results clearly show that under the condition of blocked vision, there exists a significant correlation between the patellar tendon reflex and the postural stability of stroke patients. Since the negative feedback control mechanism of the patellar tendon reflex only involves the ventral root of the spinal cord of CNS, we hypothesize that the postural stability relies more on the spinal and muscular elementary reflex when visual feedback is not available. When the visual input is available, the whole CNS response is involved, and the patellar tendon reflex becomes less dominant in controlling postural stability.

Previous studies have found that quadriceps muscle strength is associated with postural stability [23,24]. However, the patellar tendon reflex was often neglected in the study of human balance control. The experimental results implied that elderly stroke patients suffering from patellar tendon reflex disorder inherently have less balance and lower control of joint mobility, and the balance control worsens when the vision system is impaired. The sustainable postural balance is associated with multiple descending or ascending neural pathways from the brain, either directly or through other spinal interneurons. Studies have found that nearly every region of the brain is involved in balance, with the cerebellum playing a key role [33]. Stroke patients may have different levels of brain or CNS impairment, resulting in different deficits of balance control. In addition, other fundamental diseases like hypertension and diabetes associated with the stroke patients may also complicate their balance capabilities. The current study tried to single out the patellar tendon reflex and explore its correlation with postural stability in elderly stroke patients. The strong correlation under the state of closed eyes may lead to insights into clinic evaluation and treatment for rehabilitation or fall risks of elderly stroke patients. For example, to prevent fall risk, special care or attention should be given to stroke patients with impaired patellar tendon reflex when they are in a dark environment, or their vision conditions are in a poor state.

Although the results of the current study are only pertinent to elderly stroke patients, possible implications of the study may expand to patients with other neurodegenerative diseases or even elderly healthy persons, which needs more studies in the future.

## 5 Conclusions

This study confirmed that the patellar tendon reflex was associated with postural stability in stroke patients during quiet standing, and the correlation became strong and significant when visual feedback was removed. The findings demonstrated that the patellar tendon reflex as a phasic reflex affects the sensitivity of postural stability adjustments. When the visual input is blocked, the quick motor neurone discharge of the patellar tendon reflex, which only involves the ventral root of the spinal cord of CNS, plays an important role in adjusting the posture balance. These results will be helpful to the clinic strategies of rehabilitation training for elderly stroke patients with abnormal tendon reflexes.

## Appendix

1. Calculation procedure of COP PSD:

A COP coordinate signal is represented by (\text{x},y)(t)] . Its PSD is defined as follows:

(A1)
\mathop{\mathrm{lim}}\limits_{T\to \infty }\frac{1}{T}\underset{0}{\overset{T}{\int }}{| (\text{x},y)(t)| }^{2}\text{d}t=\mathop{\mathrm{lim}}\limits_{T\to \infty }\frac{1}{T}\underset{-\infty }{\overset{+\infty }{\int }}{| {F}_{\sigma }(\omega )| }^{2}\text{d}f=\underset{-\infty }{\overset{+\infty }{\int }}{S}_{\sigma }(f)\text{d}f,]

where {F}_{\sigma }(\omega )] is the Fourier transform of (x,y)(t)] , {S}_{\sigma }(f)] is the distribution of the average power spectrum (or energy) of the signal in the frequency domain, that is, the power of a unit frequency band is transformed with the frequency.

2. The calculation procedure of COP DFA:

Step 1: Calculating the cumulative deviation of time series [*v*(*i*), *t* = 1,2,…,*N*] of COP sway velocity, which is calculated from the following equation:

(A2)
v=\sqrt{{({x}_{i+1}-{x}_{i})}^{2}+{({y}_{i+1}-{y}_{i})}^{2}}/\Delta t,]

where *x*_*i*_ and *y*_*i*_ are the COP displacements in the *A/P* and *M/L* directions at sampling sequence number of *i*, and \Delta t] is the sampling interval and is 0.002 s.

(A3)
k(i)=\mathop{\sum }\limits_{i=1}^{n}{[}v(i)-\mathop{v}\limits^{\_}],]

where k(i)] was the sequence reconstruction which was divided into segments with an equal interval of *p*, and *p* was called as the scale index. The least square method was adopted to carry out 2nd order polynomial fitting for each segment, denoted as {k}_{p}(i)] .

Step 2: Calculating the fluctuation (root mean square) of the cumulative time series as follows:

(A4)
F(p)=\sqrt{\frac{1}{n}\mathop{\sum }\limits_{i=1}^{n}{(k(i)-{k}_{p}(i))}^{2}}.]

Since *v*(*i*) was a time series with self-similarity characteristics, there was a power law relationship between the mean value *F*(*p*) of the wave function and the scale index *p*, which could be expressed as the following formula:

(A5)
F(p)\propto {p}^{\alpha },]

where *α* was the scale index. When *α* > 1 (noted as *α*_2_, long-term scaling exponent) (or *α* < 1, noted as *α*_1_, short-term scaling exponent), it presented that the time series contains information related to long range (or short range).

We calculated and counted *α*_1_, *α*_2_, and their CP (critical, or crossover point).
